# Comparison of the Retention Rates of Synthetic and Natural Astaxanthin in Feeds and Their Effects on Pigmentation, Growth, and Health in Rainbow Trout (*Oncorhynchus mykiss*)

**DOI:** 10.3390/antiox11122473

**Published:** 2022-12-15

**Authors:** Wei Zhao, Yu-Cai Guo, Ming-Yan Huai, Lily Li, Chi Man, Wolf Pelletier, Han-Lin Wei, Rong Yao, Jin Niu

**Affiliations:** 1 State Key Laboratory of Biocontrol, Guangdong Provincial Key Laboratory for Aquatic Economic Animals and Southern Marine Science and Engineering Guangdong Laboratory (Zhuhai), School of Life Sciences, Sun Yat-Sen University, Guangzhou 510275, China; 2 BASF (China) Co., Ltd., Pudong, Shanghai 200137, China; 3 BASF SEA Pte Ltd., Singapore 038987, Singapore; 4 BASF East Asia Regional Headquarters Ltd., Hong Kong 999077, China; 5 BASF SE, 67056 Ludwigshafen, Germany

**Keywords:** trout, astaxanthin, coloring, antioxidant status, immunity, flesh quality

## Abstract

The coloring efficiency and physiological function of astaxanthin in fish vary with its regions. The aim of this study was to compare the retention rates of dietary astaxanthin from different sources and its effects on growth, pigmentation, and physiological function in *Oncorhynchus mykiss*. Fish were fed astaxanthin-supplemented diets (LP: 0.1% Lucantin^®^ Pink CWD; CP: 0.1% Carophyll^®^ Pink; EP: 0.1% Essention^®^ Pink; PR: 1% *Phaffia rhodozyma*; HP: 1% *Haematococcus pluvialis*), or a diet without astaxanthin supplementation, for 56 days. Dietary astaxanthin enhanced pigmentation as well as the growth of the fish. The intestinal morphology of fish was improved, and the crude protein content of dorsal muscle significantly increased in fish fed with astaxanthin. Moreover, astaxanthin led to a decrease in total cholesterol levels and alanine aminotransferase and aspartate aminotransferase activity in plasma. Fish fed on the CP diet also produced the highest level of umami amino acids (aspartic acid and glutamic acid). Regarding antioxidant capacity, astaxanthin increased Nrf2/HO-1 signaling and antioxidant enzyme activity. Innate immune responses, including lysozyme and complement systems, were also stimulated by astaxanthin. Lucantin^®^ Pink CWD had the highest stability in feed and achieved the best pigmentation, Essention^®^ Pink performed best in growth promotion and Carophyll^®^ Pink resulted in the best flesh quality. *H. pluvialis* was the astaxanthin source for achieving the best antioxidant properties and immunity of *O. mykiss*.

## 1. Introduction

*Oncorhynchus mykiss* is the most extensively reared carnivorous cold-water fish worldwide, with a production volume in 2020 of 959,600 tons [1]. The flesh and skin color of *O. mykiss* are vital evaluation criteria that determine consumer acceptance and market price. It is generally known that carotenoids, especially astaxanthin, account for the natural body color of salmonids [2]. Similar to other aquatic animals, *O. mykiss* cannot synthesize carotenoids de novo. Thus its body color depends on dietary intake. The supplementation of fish feed with carotenoids is an efficient approach to obtaining pigmentation in aquaculture [2]. Astaxanthin has been primarily used in feed for salmon, trout, and crustaceans to increase pigment deposition in the skin, muscles, or exoskeleton [3]. Moreover, dietary astaxanthin has positive effects on the growth and various physiological functions of aquatic animals (e.g., oxidation resistance, immunomodulation, stress resistance, and disease resistance) [4]. There have been significant developments in the research into, and application of, astaxanthin in aquaculture over the past few years.

Commercially available astaxanthin is primarily derived from either chemical synthesis or natural resources (e.g., the red yeast *Phaffia rhodozyma*, the bacterium *Paracoccus carotinifaciens* and the microalga *Haematococcus pluvialis*) [3,5]. Chemically synthesized astaxanthin dominates the market due to its lower production cost and better stability at higher carotenoid concentrations, accounting for 95% of the global astaxanthin market [4,6]. BASF (Badische Anilin & Sodafabrik, Ludwigshafen, Germany), DSM (Dutch State Mines, Heerlen, The Netherlands) and NHU (New Harmony Union, Shaoxing, China) are recognized as the leading global manufacturers of synthetic astaxanthin. Astaxanthin from synthetic and natural sources differs with regard to esterification and stereochemistry, thus impacting its stability, bioavailability, and physiological properties [3,7]. Furthermore, the metabolic pathways of carotenoids in fish are species-specific [6].

Previous research has focused on comparing the effects of *H. pluvialis* and synthetic astaxanthin on the body color of *O. mykiss* [8,9]. However, the effects of dietary supplementation with natural or synthetic astaxanthin on growth, oxidation resistance, innate immune regulation, and the flesh quality of *O. mykiss* remain unclear due to a lack of data. Moreover, the retention rates of synthetic and natural astaxanthin in the diet of *O. mykiss* are still unknown. Accordingly, this study was conducted to compare the effects of natural astaxanthin (e.g., *H. pluvialis* and *P. rhodozyma*) and synthetic astaxanthin (e.g., Lucantin^®^ Pink CWD (BASF, Ludwigshafen, Germany), Carophyll^®^ Pink (DSM, Heerlen, The Netherlands) and Essention^®^ Pink (NHU, Shaoxing, China)) on pigmentation, growth, oxidation resistance, innate immune regulation, flesh quality and the intestinal morphology of *O. mykiss* in aquaculture. Furthermore, this study is the first to elucidate retention rates of mainstream astaxanthin products in the diet of *O. mykiss*. The findings of this study show the effects of synthetic and natural astaxanthin on pigmentation and physiological function in *O. mykiss* and provide a valuable contribution to the development of precision functional feed for aquatic animals.

## 2. Materials and Methods

### 2.1. Astaxanthin Sources and Diet Preparation

Table 1 lists the formulation and proximate composition of the six isonitrogenous (46% crude protein) and isoenergetic (15% crude lipid) experimental diets (Control: control diet without astaxanthin supplement; LP: 0.1% Lucantin^®^ Pink CWD; CP: 0.1% Carophyll^®^ Pink; EP: 0.1% Essention^®^ Pink; PR: 1% *P. rhodozyma*; HP: 1% *H. pluvialis*) with different astaxanthin sources. The *H. pluvialis* and *P. rhodozyma* of 1% (*w*/*w*) astaxanthin concentrate came from Aiphy Biotech Co., Ltd. (Yunnan, China) and Lvjia Biotech Co., Ltd. (Nanping, China), respectively. The Lucantin^®^ Pink CWD, Carophyll^®^ Pink and Essention^®^ Pink of 10% (*w*/*w*) astaxanthin concentrate were provided by BASF (Ludwigshafen, Germany), DSM (Heerlen, The Netherlands) and NHU (Shaoxing, China), respectively. The experimental diet consisted of fish meal, soybean meal, soy protein isolate and *Tenebrio molitor* meal as protein sources, fish oil and soybean lecithin as lipid sources, corn starch and cassava starch as carbohydrate sources, supplemented with methionine and lysine to meet the nutritional requirements of *O. mykiss*. The experimental feeds were produced based on the procedures described previously [10].

### 2.2. Fish and Experimental Conditions

The *O. mykiss* used in the study originated from a local commercial company (Qinghai, China). The feeding trial was performed in the upper reaches of the Yellow River (101.0′27″ E, 36.8′22″ N) with 24 floating net cages (2.8 m × 2.7 m × 2 m). The respective experimental diets were assigned to one of four cages (30 fish per cage). Fish were fed the control diet for 2 weeks to adapt to the experimental environment. After acclimation, fish with an initial body weight of 251.04 ± 0.91 g were randomly selected for the feeding trial. The fish were fed twice a day at 8:30 a.m. and 6:30 p.m., and feeding was stopped when the fish no longer came to the surface to feed. The feeding trial lasted for 56 days. During the trial, the water temperature ranged from 11 to 15 °C, and the dissolved oxygen content in the water was not less than 6.0 mg L^−1^.

### 2.3. Sample Collection

All fish were starved for 24 h after the feeding trial. Next, all fish were anesthetized with 150 mg L^−1^ of MS-222 (Sigma-Aldrich, St. Louis, MO, USA) for counting and weighing. Three fish per cage were selected at random and then stored at −80 °C to investigate whole-body composition and astaxanthin content. Another three fish were randomly taken from each cage to collect dorsal muscle (all from the same position), and to measure color parameters. Subsequently, the dorsal muscle was stored at −80 °C to investigate proximate composition, astaxanthin concentration, amino acid, and fatty acid composition. The intestines of these three fish were then soaked in 4% paraformaldehyde for morphology analysis. Afterward, three fish from each cage were used to measure pigmentation in fresh fillets at the horizontal position of the lateral line. Another six fish per cage were selected to collect plasma, which was stored at −80 °C until the analysis of biochemical parameters. Lastly, the livers of these six fish were removed and frozen in liquid nitrogen to determine enzyme activity and to perform Real-Time Quantitative PCR (qPCR) as well as Western Blotting analysis.

### 2.4. Nutritional Composition, Pigmentation, Astaxanthin Content and Morphological Analysis of Samples

The proximate compositions of the experimental diets, whole body, and dorsal muscle, including moisture, crude lipid, and crude protein content, were analyzed using a standard method of AOAC [11]. The amino acid and fatty acid compositions in the dorsal muscles were quantified using the method presented in an earlier study [12].

A hand-held colorimeter (CR-10 plus, Konica Minolta, Tokyo, Japan) was used to measure the color parameters of the dorsal muscles. L*, a* and b* represent lightness, redness, and yellowness, respectively. Spectrophotometry was used to determine the astaxanthin content in the dorsal muscles and whole body and test diets according to a previously reported method [13]. Color differences in fresh fillets were quantified using color cards (BASF Salmon Color Fan, BASF, Ludwigshafen, Germany). The color values of fresh fillets were examined independently by two researchers. For each experimental diet, the results from both researchers were used to calculate an average value for coloring ability.

Histological observation after hematoxylin and eosin staining was performed in accordance with the procedures presented in an earlier study [14]. Gut samples were fixed in 4% paraformaldehyde and then dehydrated in a graded ethanol series (75%, 4 h; 85%, 2 h; 90%, 2 h; 95%, 1 h; 100%, 1 h). Then, the gut samples were immersed in a mixed solution of ethanol (100%) and xylene with a volume ratio of 1:1 for 30 min, followed by immersion in xylene solution for 30 min. After dehydration, the gut samples were embedded in paraffin. Sections (5 mm thick) of the midgut and liver were obtained with a rotary microtome and stained with hematoxylin and eosin. Finally, the sections were observed and photographed using an optical microscope (Leica DMLB, Leica, Wetzlar, Germany).

### 2.5. Plasma and Antioxidant Parameters and qPCR Analysis

Alanine aminotransferase (ALT) (C009-2-1) and aspartate aminotransferase (AST) (C010-2-1) activity levels, as well as total cholesterol (T-CHO) (A111-1-1) and lysozyme (Lyz) (A050-1-1) levels in plasma, were obtained using commercial assay kits (Jiancheng Bioengineering Institute, Nanjing, China), in accordance with the instructions.

Superoxide dismutase (SOD) (A001-1-1) activity, malondialdehyde (MDA) (A003-1-2) content and total antioxidant capacity (T-AOC) (A015-2-1) were examined in liver tissue using commercial assay kits (Jiancheng Bioengineering Institute), in accordance with the instructions.

qPCR was performed in accordance with the procedure described in an earlier study [15]. The gene-specific primers are shown in Table 2. β-actin was set as the reference gene. The relative translation levels of the respective gene were obtained based on the 2^−ΔΔCT^ method.

### 2.6. Calculations and Statistical Analysis

Growth parameters were calculated according to the equation described previously [15]. Condition factor (CF) was obtained as follows: CF (g/cm^3^) = 100 × (body weight in g)/(body length in cm)^3^.

The data are expressed as means ± standard error (SE) and were subjected to one-way ANOVA analysis using SPSS 20.0 software. The normality and homogeneity of data were analyzed using the Kolmogorov-Smirnov test and Levene’s test, respectively. Subsequently, differences between the experimental diets were determined with Duncan’s multiple-range test. The data were considered to have significant differences at *p* < 0.05.

## 3. Results

### 3.1. Pigmentation and Astaxanthin Content

**Figure 1:**
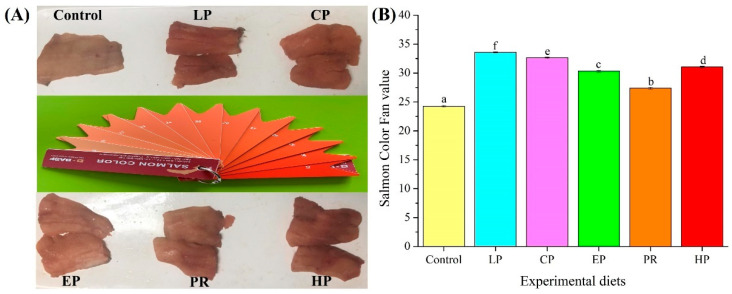
Comparison of pigmentation in the dorsal muscle of *Oncorhynchus mykiss* fed on an experimental diet for 56 days ((**A**) Visual color inspection; (**B**) Salmon color fan value). The color value of fresh fillet in *Oncorhynchus mykiss* fed on experimental diets. Values are presented as mean ± SE, n = 12. Different letters represent statistical differences (*p* < 0.05).

Visual color inspection shows that the diets supplemented with astaxanthin significantly improved the pigmentation of abdominal muscle (Figure 1A). The BASF salmon color fan cards were used to quantify the results of visual color inspections, with higher color fan values indicating higher redness values. The BASF salmon color fan value in the control group was significantly lower than that in the LP, CP, EP, PR, and HP diet groups (*p* < 0.05) (Figure 1B). The color fan value of synthetic astaxanthin diets (LP and CP) was also significantly higher than that of natural astaxanthin diets (PR and HP) (*p* < 0.05), indicating that synthetic astaxanthin (LP and CP) supplementation showed greater efficiency than natural astaxanthin (PR and HP) in improving the pigmentation of abdominal muscle in *O. mykiss*.

Colorimetric analysis shows that the color parameters (L*, a*, and b* values) of abdominal muscle were affected by dietary astaxanthin (*p* < 0.05) (Table 3). Fish fed with astaxanthin-supplemented diets (LP, CP, EP, PR, and HP) had a significantly lower lightness (L*) value than those fed the control diet (*p* < 0.05). In contrast, higher redness (a*) values in abdominal muscle were obtained in fish that were fed diets supplemented with astaxanthin (LP, CP, EP, PR, and HP) (*p* < 0.05). Meanwhile, fish fed with synthetic astaxanthin-supplemented diets (LP, CP, and EP) showed significantly higher a* values than those fed on diets supplemented with natural astaxanthin (PR and HP) (*p* < 0.05). Likewise, higher yellowness (b*) values were obtained in fish fed on diets supplemented with synthetic astaxanthin (LP, CP, and EP) (*p* < 0.05), while there was no significant difference between natural astaxanthin (PR and HP) and the control diet (*p* > 0.05).

Fish fed on diets supplemented with astaxanthin (LP, CP, EP, PR, and HP) had higher astaxanthin content in their abdominal muscle and whole body than those fed the control diet (*p* < 0.05) (Table 4). Synthetic astaxanthin (LP, CP, and EP) was superior to natural astaxanthin (PR and HP) in promoting astaxanthin accumulation in abdominal muscles and in the whole body (*p* < 0.05).

### 3.2. Retention Rates of Astaxanthin in Feeds

As shown in Table 5, the astaxanthin retention rate of the LP diet was the highest after being extruded into pellets, followed by HP, PR, CP, and EP. When all experimental diets were stored for one month, the astaxanthin retention rate was the highest in the LP diet, followed by EP, HP, PR, and CP. Similarly, when the experimental diets were stored for two months, the astaxanthin retention rate was the highest in the LP diet, followed by EP, CP, HP, and PR. The results indicated that the retention rates of synthetic astaxanthin (LP, CP, and EP) in feeds were better than that of natural astaxanthin (PR and HP) after two months of storage, and Lucantin^®^ Pink CWD showed the lowest extrusion losses and the highest stability during storage.

### 3.3. Biological Parameters

There were no significant differences in survival rate (SR) and CF between the experimental diets (*p* > 0.05). Higher final body weight (FBW), weight gain rate (WGR) and specific growth ratio (SGR) were obtained in fish fed on diets supplemented with astaxanthin (LP, CP, EP, PR, and HP) (*p* < 0.05). Meanwhile, fish fed on the EP and HP diets showed better FBW and WGR than the other astaxanthin groups (*p* < 0.05). The feed conversion ratio (FCR) of fish fed on the LP and EP diets was significantly lower than that of fish fed with the control diet (*p* < 0.05), but there was no significant difference between the astaxanthin groups (*p* > 0.05) (Table 6).

### 3.4. Whole Body and Muscle Composition Analysis

As shown in Table 7, the moisture content in the whole body and dorsal muscle was not affected by the experimental diets (*p* > 0.05). Astaxanthin groups (LP, CP, EP, PR, and HP) showed a significant decrease in whole-body crude lipid content and an increase in dorsal muscle crude protein content (*p* < 0.05), whereas there was no significant effect of the experimental diets on the crude lipid content of dorsal muscle (*p* > 0.05). Except for PR, fish that were fed diets supplemented with astaxanthin (LP, CP, EP and HP) had an obviously higher level of crude protein in the whole body compared to those fed the control diet (*p* < 0.05).

### 3.5. Amino Acid and Fatty Acid Composition Analysis

Higher levels of non-essential amino acids (NEAA) and total amino acids (TAA) in the dorsal muscle were obtained in fish fed on the CP, PR, and HP diets, but there were no obvious differences between all treatments (*p* > 0.05). Fish fed on the CP diet had higher essential amino acid (EAA) levels than those fed the EP diet (*p* < 0.05), but there was no significant difference with the other diet groups (*p* > 0.05). Moreover, the CP diet produced the highest level of umami amino acids (including aspartic acid (Asp) and glutamic acid (Glu)) of all experimental diets (Table 8).

**Figure 2:**
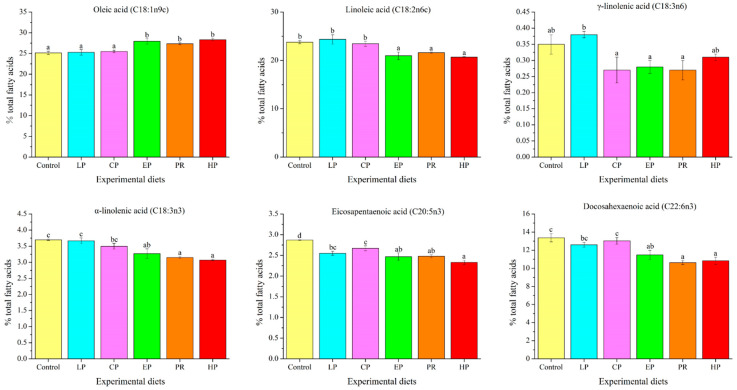
Long-chain fatty acid levels in the abdominal muscle of *Oncorhynchus mykiss* fed with the experimental diets for 56 days. Values are presented as mean ± SE, n = 4. The superscript letters indicate a significant difference at *p* < 0.05.

The long-chain fatty acid content of abdominal muscle was significantly affected by dietary astaxanthin (*p* < 0.05) (Figure 2). The oleic acid level in the control, LP, and CP diet groups was remarkably lower compared to the EP, PR, and HP diet groups (*p* < 0.05), whereas the linoleic acid (LA) level was contrary to that of oleic acid. The γ-linolenic acid (γ-LNA) level was not affected by the experimental diets (*p* > 0.05). The levels of α-linolenic acid (α-LNA) and docosahexaenoic acid (DHA) in the PR and HP diet groups were significantly lower compared to the control, LP and CP diet groups (*p* < 0.05), but there was no significant difference from the EP diet group (*p* > 0.05). It is noteworthy that the astaxanthin-supplemented diets reduced the level of eicosapentaenoic acid (EPA) in abdominal muscle (*p* < 0.05).

### 3.6. Morphological Analysis

**Figure 3:**
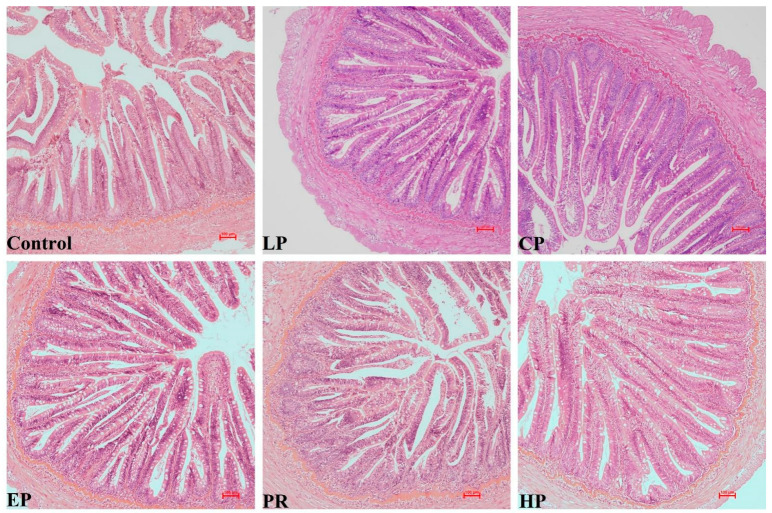
The intestinal histology of *Oncorhynchus mykiss* was fed with experimental diets for 56 days. Magnification 200×.

No remarkable histological changes were observed in the middle intestine under any dietary regimen (Figure 3).

**Figure 4:**
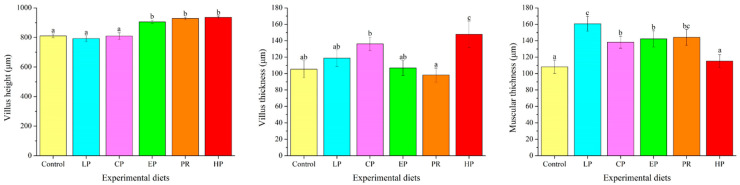
Effects of different diet treatments on the intestinal morphometry of *Oncorhynchus mykiss*. Values are presented as mean ± SE, n = 12. The superscript letters indicated significant differences at *p* < 0.05.

Examination of the intestinal morphology showed that villus height in the EP, PR and HP diet groups was remarkably higher than in the control, LP and CP diet groups (*p* < 0.05). The highest value of villus thickness was obtained in the HP diet group and was significantly higher than in the other diet groups (*p* < 0.05). Fish fed the LP, CP, EP and PR diets had higher mucosal thickness than those fed the control and HP diets (*p* < 0.05) (Figure 4).

### 3.7. Plasma Biochemical Parameters

**Figure 5:**
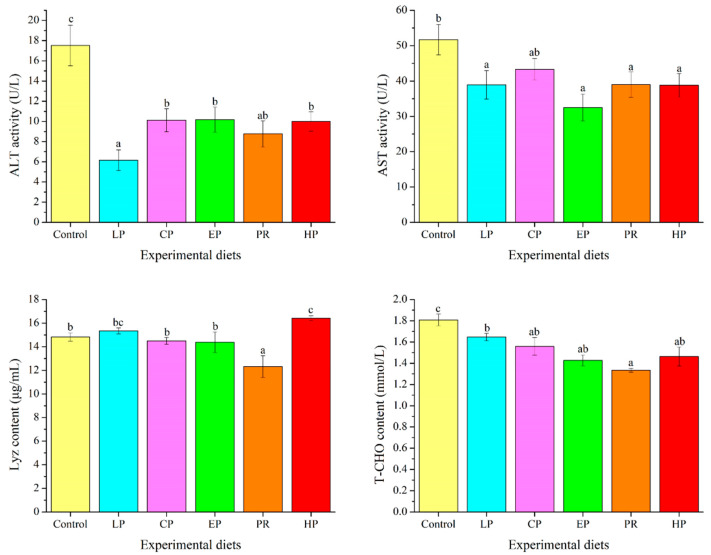
Effects of different diet treatments on the plasma parameters of *Oncorhynchus mykiss*. Values are presented as mean ± SE, n = 12. The superscript letters indicated significant differences at *p* < 0.05.

ALT activity and T-CHO levels in the astaxanthin-supplemented groups (LP, CP, EP, PR, and HP) were remarkably lower than in the control group (*p* < 0.05). Fish fed on the LP, EP, PR, and HP diets had lower AST activity than those fed on the control diet (*p* < 0.05). Compared with the control group, Lyz content was significantly enhanced by the HP diet and decreased by the PR diet (*p* < 0.05) (Figure 5).

### 3.8. Antioxidant Parameters in the Liver

**Figure 6:**
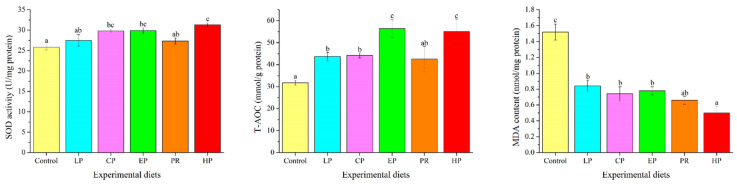
Effects of different diet treatments on antioxidant parameters in *Oncorhynchus mykiss*. Values are presented as mean ± SE, n = 12. The superscript letters indicate significant differences at *p* < 0.05.

Antioxidant parameters were significantly affected by dietary astaxanthin (*p* < 0.05) (Figure 6). MDA levels in the livers of the astaxanthin-supplemented groups (LP, CP, EP, PR, and HP) were significantly lower than in the control group (*p* < 0.05). Fish fed on the CP, EP and HP diets had higher SOD activity than those fed on the control diet (*p* < 0.05), but there was no obvious difference from the control, LP and PR diets (*p* > 0.05). Except for PR, fish fed on the diets supplemented with astaxanthin (LP, CP, EP and HP) had significantly higher T-AOC values than those fed on the control diet (*p* < 0.05).

**Figure 7:**
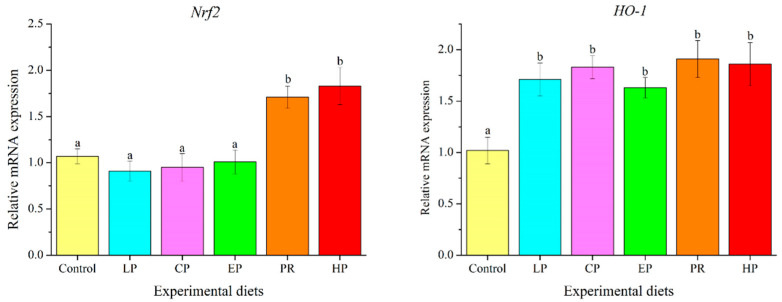
Effects of different diet treatments on the expression of antioxidant-related genes in the liver of *Oncorhynchus mykiss*. Values are presented as mean ± SE, n = 12. The superscript letters indicated significant differences at *p* < 0.05.

Fish fed with natural astaxanthin (PR and HP) showed significantly higher mRNA levels of NF-E2-related nuclear factor 2 (*Nrf2*) than those fed the control diet or synthetic astaxanthin (LP, CP, and EP) (*p* < 0.05). The transcription level of heme oxygenase-1 (*HO-1*) was significantly up-regulated by diets supplemented with astaxanthin (*p* < 0.05) (Figure 7).

### 3.9. Expression Analysis of Immune-Related Genes in Liver

**Figure 8:**
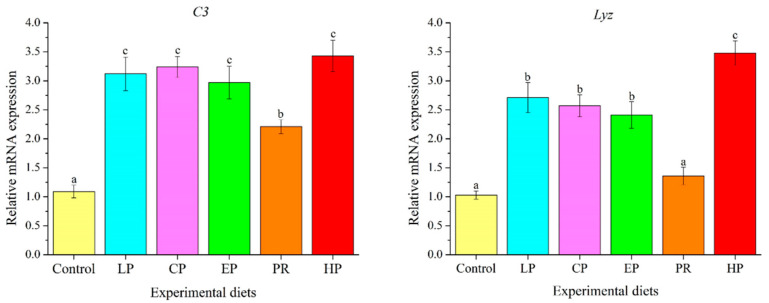
Effects of different diet treatments on the expression of immune-related genes in the liver of *Oncorhynchus mykiss*. Values are presented as mean ± SE, n = 12. The superscript letters indicated significant differences at *p* < 0.05.

As depicted in Figure 8, mRNA levels of complement 3 (*C3*) in the astaxanthin-supplemented groups (LP, CP, EP, PR, and HP) were remarkably higher than in the control group (*p* < 0.05). Except for PR, mRNA levels of *Lyz* in the LP, CP, EP and HP diets were significantly higher than in the control group (*p* < 0.05).

## 4. Discussion

### 4.1. Pigmentation and Astaxanthin Accumulation

A well-established function of astaxanthin in aquatic animals is the enhancement of skin and flesh coloration. For *O. mykiss*, attractive flesh color is critical to commercial value since it is an important criterion by which consumers judge product quality (e.g., freshness, nutritional value, and flavor), which ultimately affects market price. In this study, the supplementation of astaxanthin in the diet significantly enhanced the pigmentation of dorsal muscle in *O. mykiss* as assessed by visual inspection and the photometric measurement of color variables. Astaxanthin supplementation decreased lightness (L*) and increased redness (a*) values, as well as the BASF Salmon Color Fan score for fresh fillets, which is consistent with previous findings [16]. Likewise, the existing research has demonstrated that adding astaxanthin to the diet obviously enhanced the color of skin and flesh in fish, including *Pagrus pagrus* [17], *Larimichthys croceus* [18] and *Salmo salar* [19]. Furthermore, this study suggests that synthetic astaxanthin, particularly Lucantin^®^ Pink CWD, is superior to natural astaxanthin in pigmenting muscle in *O. mykiss*, based on the comparison of a* and b* values. The above results are fully consistent with previous findings in *O. mykiss* [8,9], where natural astaxanthin was less efficient in muscle pigmentation than synthetic astaxanthin. Consistent with color parameters, astaxanthin supplementation significantly increased concentrations of this carotenoid in the dorsal muscle and in the whole body. It is noteworthy that natural astaxanthin exists primarily in esterified form, while synthetic astaxanthin exists in its free form [3]. Esterified astaxanthin needs to be hydrolyzed during digestion before it becomes bioavailable, which may be one reason for the impaired deposition of natural astaxanthin in the tissue of *O. mykiss* and in *S. salar* [20]. Furthermore, the cell walls of microalgae and yeast result in poor digestibility, which may also decrease the bioavailability of natural astaxanthin. The findings of this study reveal that synthetic astaxanthin produces more effective coloration than natural astaxanthin in *O. mykiss*.

### 4.2. Retention Rate of Astaxanthin in Feed

Astaxanthin, in its esterified form, is commonly considered more stable than free astaxanthin. However, esterified astaxanthin is susceptible to chemical degradation and oxidation due to its highly unsaturated structure, which results in the loss of nutritional and biological activity [21,22]. An interesting result of this study is that the retention rate of synthetic astaxanthin in feed was better than that of natural astaxanthin after diet preparation and storage. This may be due to the fact that commercially synthesized astaxanthin is usually microencapsulated and stabilized with antioxidants. The microencapsulation of astaxanthin also significantly improves its bioavailability [22]. In this study, synthetic astaxanthin was superior to natural astaxanthin in terms of growth promotion and coloring efficiency: this may be closely linked to its production process, which improves the bioavailability of astaxanthin. We conclude that Lucantin^®^ Pink CWD has the best retention rate of astaxanthin in feed.

### 4.3. Growth Parameters and Intestinal Morphology

Carotenoids account for the pigmentation of fish skin and flesh, but there is an increasing interest in other biological functions, such as promoting growth, antioxidant activity, and immune regulation in aquatic animals. Early on, astaxanthin was shown to enhance nutrient utilization by mediating the intermediary metabolism, which ultimately resulted in increased growth performance [23]. In this study, the addition of astaxanthin to the diet produced beneficial effects on growth and feed utilization in commercial-size *O. mykiss*. This is in accordance with previous findings in *O. mykiss* fed on diets that had been supplemented with different sources and concentrations of astaxanthin [24,25]. Moreover, previous research demonstrated that supplementation with astaxanthin significantly improved the growth performance of *Pseudosciaena crocea* [26], *Gadus morhua* [27] and *Trachinotus ovatus* [28]. In contrast, some studies have suggested that dietary astaxanthin does not facilitate fish growth, including *Paralichthys olivaceus* [29], *Larimichthys croceus* [25], *Sparus aurata* [30], as well as *O. mykiss* [31,32]. The effect of dietary astaxanthin on the growth performance of aquatic animals is closely related to the development stage, culturing environment, differences between species, astaxanthin source and feeding duration [4]. Thus far, the effect of astaxanthin on fish growth remains controversial, and the mechanisms by which astaxanthin may affect growth warrant further study.

Examination of intestinal morphology is a useful way of evaluating health and the functional status of the fish’s gut system, which is mainly assessed by measuring villi length, villi thickness and muscular thickness [33]. In fish, the intestine is the principal organ responsible for digesting and absorbing nutrients from the diet. Thus, digestive function is closely correlated with intestinal development [34]. Earlier studies reported that an increase in villi length and villi thickness implies an enlargement of the surface area and, consequently, improved absorption of nutrients [14,35]. Muscular thickness is closely correlated with the peristaltic capacity of the intestine. In general, an increase in muscular thickness may facilitate intestinal peristalsis, which is beneficial to the transport and absorption of nutrients in the intestine [36]. In this study, astaxanthin had positive effects on the intestinal morphology of *O. mykiss* (e.g., increasing villi length, villi thickness or muscular thickness). Similar observations were found in the intestine of *T. ovatus*, where dietary *H. pluvialis* enhanced villi length in the midgut [37]. The observed positive effects of astaxanthin on villi length, villi thickness and muscular thickness may help to improve the absorption and utilization of nutrients, which may lead to better growth performance in *O. mykiss*.

### 4.4. Flesh Quality

In this study, astaxanthin-supplemented diets significantly changed the nutritional composition of dorsal muscle and the whole body of *O. mykiss*. Likewise, in a study with *P. pagrus*, it was shown that synthetic astaxanthin reduced the crude lipid content of the whole body [38]. The findings of both studies suggest that the perivisceral lipid content is reduced as a result of astaxanthin intake. Besides, lipids are the main energy source of fish. In this study, astaxanthin-supplemented diets decreased the crude lipid content of the whole body. A possible reason for this result is that astaxanthin stimulates lipid utilization, making it available as an energy source and ultimately improving growth performance [38]. Moreover, higher lipid utilization may save protein for energy metabolism and store excess protein in muscles and the whole body. However, in contrast to our results, higher lipid levels in the whole body and flesh were reported when fish were fed with an astaxanthin-supplemented diet, including *S. salar* [39], *P. crocea* [26] and *P. pagrus* [6]. Accordingly, the effects of astaxanthin from different sources and administered at different dosage levels on the composition of the whole body, flesh and liver remain controversial and contradictory, and further research is needed to clarify the mechanism of dietary astaxanthin on lipids in fish.

The impact on fatty acid composition indicates that fish fed on the EP, PR and HP diets had a decrease in long-chain unsaturated fatty acids (LA, LNA and DHA) in their abdominal muscles. EPA levels also decreased in all astaxanthin groups. This suggests that astaxanthin supplementation may inhibit the biosynthesis of n-3 and n-6 unsaturated fatty acids in the muscle tissue of *O. mykiss*, thus revealing a possible decrease in elongation and desaturation activity. In contrast, feeding synthetic astaxanthin to *P. pagrus* increased EPA and DHA levels in the liver [38]. Earlier studies also suggested that astaxanthin supplementation had no effect on the composition of fatty acids in the liver or in fillets of *P. olivaceus* [29], *P. pagrus* [6] or *O. mykiss* [40]. The above contradictory results are difficult to explain. The regulatory mechanism of astaxanthin on the fatty acid composition of fish remains to be clarified. In general, Carophyll^®^ Pink yielded moderately higher levels of long-chain unsaturated fatty acids in dorsal muscle than the other astaxanthin sources.

The amino acid compositions of the dorsal muscles were slightly affected by the different astaxanthin sources. The amino acid composition of muscle shows a minor response to dietary composition compared with the fatty acid profile, probably due to the lower protein synthesis rate in fish muscle [12]. In this study, dietary astaxanthin did not positively affect the total levels of EAA, NEAA and amino acids in dorsal muscle. Amino acids provide primary taste properties: ASP and Glu are responsible for the umami and savory flavors in the meat, whereas Ala and Gly have sweet tastes [41,42]. Thus, ASP, Glu, Ala and Gly are considered the primary indicators of flavor quality [43]. The findings of this study suggested that fish-fed CP and PR diets increased the ASP and Glu content in the dorsal muscle. The addition of CP and PR to the diet increases the umami flavor of fish flesh, thus making the product more popular with consumers.

### 4.5. Plasma Biochemical Indices

Hematological parameters serve as vital indicators in evaluating physiological and pathological changes in fish and are often used in disease diagnosis and the evaluation of nutritional status [44,45]. ALT and AST activity in plasma is used to diagnose liver integrity and function [46,47]. This study reveals that dietary astaxanthin down-regulates the activity of ALT and AST in plasma, which is consistent with previous observations in other fish species fed on diets supplemented with different sources and concentrations of astaxanthin [46,48]. It is noteworthy that fish in aquaculture are sensitive to environmental stressors and infections [14]. Accordingly, supplementing fish feed with astaxanthin is considered helpful in relieving environmental stress and protecting the liver, in turn improving fish health in cage culture [14].

Changes in plasma cholesterol levels have been commonly used to evaluate whether liver function or lipid metabolism is abnormal [49]. In this study, dietary astaxanthin supplementation led to a decrease in plasma T-CHO, revealing the anti-hyperlipidemic potential of astaxanthin. Our findings are consistent with those of Lim et al. [46] and Sheikhzadeh et al. [47], in which dietary astaxanthin or *H. pluvialis* clearly down-regulated serum T-CHO levels in fish. Astaxanthin is capable of enhancing the clearance of endogenous cholesterol to alleviate hyperlipidemia and thus relieve stress in fish, which may be attributed to its antioxidant properties [46]. Previous research hypothesized that the functional mechanism of astaxanthin against hyperlipidemia is primarily regulated by the peroxisome proliferator-activated receptor (PPARs) [50].

### 4.6. Antioxidation Property

Nrf2, a cytoprotective transcription factor, is capable of mediating the transcription level of genes involved in cellular antioxidant and anti-inflammatory defense in response to oxidative stress by binding to antioxidant response elements [51]. HO-1, a crucial target gene of Nrf2, can be rapidly activated by oxidative stress and exerts a cytoprotective role against oxidative stress-induced cytotoxicity [52,53]. Earlier studies revealed that astaxanthin protects cells by inducing antioxidant activity through the activation of Nrf2/HO-1 signaling [54,55]. Likewise, this study finds that supplementation with natural astaxanthin up-regulated the mRNA level of *Nrf2*. Moreover, dietary synthetic astaxanthin up-regulated the mRNA levels of *HO-1* but did not induce *Nrf2* transcription. This result may indicate that HO-1 is regulated by other activators besides Nrf2. Supporting our findings, astaxanthin can induce the transcription of *Nrf2* and *HO-1* to strengthen cellular defense against oxidative stress [54]. Consistent with the above findings, it has been shown that synthetic astaxanthin and *H. pluvialis* may relieve oxidative stress and enhance oxidation resistance in *T. ovatus* by activating the Nrf2/HO-1 signal pathway [28,37].

SOD, the first line of antioxidant defense against free radical toxicity in cells, is capable of converting superoxide (•O_2_^−^) to hydrogen peroxide (H_2_O_2_) through disproportionation, which helps to eliminate free radicals and maintain the redox balance in cells [10]. As a product of lipid peroxidation, the MDA level indirectly reflects the degree of the cell damage caused by free radicals. T-AOC is a key bioindicator for evaluating the activity of all antioxidants (enzymatic and non-enzymatic) in the body [10]. This study demonstrates that dietary astaxanthin may have beneficial effects on SOD activity and T-AOC (except for the PR diet). Moreover, dietary astaxanthin led to considerably lower MDA levels. Similar findings have been observed in *O. mykiss* [47], *P. croceaas* [26], *Symphysodon* spp. [56] and *T. ovatus* [37] as a direct result of astaxanthin or *H. pluvialis* supplementation. The results of this study demonstrate that adding astaxanthin to the diet has beneficial effects on the antioxidant system of fish, whereas the effect of *P. rhodozyma* was weaker than that of *H. pluvialis* and synthetic astaxanthin. The primary optical isomer of astaxanthin in *H. pluvialis* was (3S,3′S), and in *P. rhodozyma*, it was (3R,3′R). The synthetic astaxanthin was composed of three optical isomers, including (3S,3′S), (3R,3′S), and (3R,3′R) at 1:2:1. Astaxanthin in the form of (3S,3′S) exhibited optimal antioxidant and anti-aging activities in vivo and in vitro, followed by (3R,3′S) and (3R,3′R) [57]. This may explain why *P. rhodozyma* improved antioxidant properties less than *H. pluvialis* and synthetic astaxanthin, and this may correlate to the differences in astaxanthin structure.

### 4.7. Immunological Characteristics

Lysozyme is a key antimicrobial protein in the innate immune system of teleost fish, which exerts bacteriolytic activity through synergistic interaction with the complement system and phagocytes [58]. Complements bind pathogenic microorganisms and are then recognized by phagocytes with complement receptors [59]. Accordingly, lysozyme and complements have been widely used as biomarkers to evaluate the innate immune status of fish [60]. Supporting the findings of this study, earlier studies revealed that dietary astaxanthin had a positive effect on lysozyme activity and the complement system in *T. ovatus* [37], *P. crocea* [26] and *Channa argus* [61]. Consistent with the results on antioxidant properties, the improvement in innate immune function in fish due to *P. rhodozyma* was also less than that due to *H. pluvialis* and synthetic astaxanthin. The improvement of the immune system due to astaxanthin is closely correlated with its antioxidant properties [46]. Thus, the weaker antioxidant properties of *P. rhodozyma* also attenuate its stimulation of the innate immune system, compared with *H. pluvialis* and synthetic astaxanthin.

## 5. Conclusions

Astaxanthin, both natural and synthetic, increased growth performance, muscle coloration, antioxidant capacity and innate immune response, and improved intestinal morphology in *O. mykiss.* Synthetic Lucantin^®^ Pink CWD had the best stability and achieved the best pigmentation, Essention^®^ Pink performed best in growth promotion, and Carophyll^®^ Pink resulted in the best flesh quality (omega-3 content). The natural astaxanthin derived from *H. pluvialis* was the best astaxanthin for achieving antioxidant properties and innate immune response. Therefore, the precision functional feed can be produced based on the coloring ability and physiological functions of different astaxanthin sources.

## Figures and Tables

**Table 1:** Composition and nutrient levels of the experimental diets (% dry matter).

Ingredients	Control	LP	CP	EP	PR	HP
Fish meal	43	43	43	43	43	43
Soybean meal	5	5	5	5	5	5
Soy protein isolate	10	10	10	10	10	10
Corn starch	10.7	10.6	10.6	10.6	9.7	9.7
*Tenebrio molitor* meal	8	8	8	8	8	8
Cassava starch	5	5	5	5	5	5
Fish oil	12	12	12	12	12	12
Soybean lecithin	2	2	2	2	2	2
Ca (H_2_PO_4_)_2_	1	1	1	1	1	1
Vitamin premix ^a^	1	1	1	1	1	1
Mineral premix ^b^	1	1	1	1	1	1
Choline	0.5	0.5	0.5	0.5	0.5	0.5
Vitamin C	0.5	0.5	0.5	0.5	0.5	0.5
DL-Met	0.2	0.2	0.2	0.2	0.2	0.2
Lys-HCL (99%)	0.1	0.1	0.1	0.1	0.1	0.1
Lucantin Pink CWD (10%) ^c^	0	0.1	0	0	0	0
Carophyll Pink (10%) ^d^	0	0	0.1	0	0	0
Essention Pink (10%) ^e^	0	0	0	0.1	0	0
*Phaffia rhodozyma* (1%) ^f^	0	0	0	0	1	0
*Haematococcus Pluvialis* (1%) ^g^	0	0	0	0	0	1
Total	100	100	100	100	100	100
Nutrient levels ^h^						
Crude protein	46.47	46.75	46.76	46.02	46.45	46.47
Crude lipid	15.46	16.12	15.66	15.68	15.49	15.37
Moisture	9.33	9.58	9.61	9.77	9.16	9.39

^a^ Multi-vitamin (kg^−1^ diet): Vitamin B1 30 mg; Vitamin B2 60 mg; Vitamin B6 20 mg; Nicotinic acid 200 mg; Calcium pantothenate 100 mg; Inositol 100 mg; Biotin 2.5 mg; Folic acid 10 mg; Vitamin B12 0.1 mg; Vitamin K3 40 mg; Vitamin A 10000IU; Vitamin D3 2000IU; Vitamin E 160IU. ^b^ Multi-mineral (kg^−1^ diet): MgSO_4_∙7H_2_O 1090 mg; KH_2_PO_4_ 932 mg; NaH_2_PO_4_∙2H_2_O 432 mg; FeC_6_H_5_O_7_∙5H_2_O 181 mg; ZnCl_2_ 80 mg; CuSO_4_∙5H_2_O 63 mg; AlCl_3_∙6H_2_O 51 mg; MnSO_4_∙H_2_O 31 mg; KI 28 mg; CoCl_2_∙6H_2_O 6 mg; Na_2_SeO_3_∙H_2_O 0.8 mg. ^c^ Lucantin Pink CWD containing 10% astaxanthin purchased from BASF (Baden Aniline and Soda Factory), Shanghai, China. ^d^ Carophyll Pink containing 10% astaxanthin purchased from DSM (Dutch State Mines), Shanghai, China. ^e^ Essention Pink containing 10% astaxanthin purchased from NHU (New Harmony Union), Xinchang, Zhejiang Province, China. ^f^ *Phaffia rhodozyma* containing 1% astaxanthin purchased from Lujia Biotech Co., Ltd., Nanping, Fujian Province, China. ^g^ The *Haematococcus Pluvialis* powder containing 1% astaxanthin was purchased from Aiphy Biotech Co., Ltd., Yunnan, China. ^h^ Measured values.

**Table 2:** Primer information of Real-time fluorescent quantitative PCR.

Gene	Primer Sequence (5′ to 3′)	Genbank No.
*Nrf2*-F	GCAGAGGTCTGCCCACCTGAAT	HQ916348.1
*Nrf2*-R	GCCACAAGGCAGGGTGACACTT	
*HO-1*-F	CGCCTACACCCGTTACCTAG	AF099079.2
*HO-1*-R	CTCTCCGCTGCTTAACCCAA	
*Lyz*-F *Lyz*-R	GAAACAGCCTGCCCAACT GTCCAACACCACACGCTT	AF452171.1
*C3*-F	GGCCAGTCCCTGGTGGTTA	XM_036955530.1
*C3*-R	GGTGGACTGTGTGGATCCGTA	
*β-actin*-F	TACAACGAGCTGAGGGTGGC	AJ438158.1
*β-actin*-R	GGCAGGGGTGTTGAAGGTCT	

**Table 3:** The color parameters of dorsal muscle in *Oncorhynchus mykiss* fed experimental diets.

	Lightness (L*)	Redness (a*)	Yellowness (b*)
Control	39.51 ± 1.11 ^b^	7.21 ± 0.74 ^a^	7.98 ± 0.39 ^a^
LP	31.76 ± 0.99 ^a^	12.79 ± 0.58 ^c^	9.74 ± 0.62 ^bc^
CP	33.59 ± 0.75 ^a^	12.17 ± 0.56 ^c^	10.43 ± 0.56 ^c^
EP	33.57 ± 0.78 ^a^	12.37 ± 0.61 ^c^	10.18 ± 0.42 ^c^
PR	34.43 ± 1.17 ^a^	9.22 ± 0.59 ^b^	7.74 ± 0.35 ^a^
HP	34.02 ± 0.85 ^a^	9.78 ± 0.72 ^b^	8.40 ± 0.45 ^ab^

Values are presented as mean ± SE, n = 12. Superscript letters in the same column indicate a significant difference at *p* < 0.05.

**Table 4:** Astaxanthin content (mg/kg) in the whole body and dorsal muscle of *Oncorhynchus mykiss* fed with the experimental diets.

	Control	LP	CP	EP	PR	HP
Whole body						
Astaxanthin content	2.76 ± 0.28 ^a^	6.32 ± 0.42 ^c^	5.91 ± 0.49 ^c^	5.79 ± 0.55 ^c^	4.01 ± 0.54 ^b^	4.42 ± 0.66 ^b^
Dorsal muscle						
Astaxanthin content	1.59 ± 0.08 ^a^	5.65 ± 0.44 ^c^	5.22 ± 0.46 ^c^	4.93 ± 0.22 ^c^	2.93 ± 0.24 ^b^	3.23 ± 0.43 ^b^

Values are presented as mean ± SE, n = 12. Superscript letters in the same row indicate a significant difference at *p* < 0.05.

**Table 5:** Retention rates of astaxanthin in feeds.

Item	Control	LP	CP	EP	PR	HP
Astaxanthin content in feeds before extrusion (mg/kg)						
	5.02	94.35	104.02	96.13	96.32	97.03
Astaxanthin content in feeds after extrusion (mg/kg)						
	4.93	92.34	100.02	91.12	93.31	94.21
Astaxanthin retention in feeds after extrusion (%)						
	98.21	97.87	96.15	94.79	96.88	97.09
Astaxanthin content in feeds after 1-month (mg/kg)						
	4.62	86.32	90.02	85.12	84.28	86.57
Astaxanthin retention in feed after 1-month (%)						
	93.71	93.48	90.00	93.41	90.32	91.89
Astaxanthin content in feeds after 2-month (mg/kg)						
	4.14	76.00	76.52	72.96	67.06	72.36
Astaxanthin retention in feed after 2-month (%)						
	89.66	88.04	85.00	85.71	79.57	83.59

**Table 6:** The growth performance of *Oncorhynchus mykiss* fed with the experimental diets.

	Control	LP	CP	EP	PR	HP
IBW (g)	251.35 ± 1.27	249.92 ± 0.63	251.22 ± 1.12	251.43 ± 0.98	250.46 ± 0.87	251.86 ± 0.58
FBW (g)	618.01 ± 5.20 ^a^	649.80 ± 6.18 ^b^	652.32 ± 4.44 ^b^	670.39 ± 3.86 ^c^	651.98 ± 6.21 ^b^	672.71 ± 7.10 ^c^
WGR (%)	145.92 ± 3.05 ^a^	160.04 ± 2.44 ^b^	159.67 ± 1.61 ^b^	166.66 ± 2.53 ^c^	160.33 ± 2.75 ^b^	165.22 ± 5.89 ^bc^
SGR (%/d)	1.00 ± 0.01 ^a^	1.06 ± 0.01 ^b^	1.06 ± 0.01 ^b^	1.09 ± 0.01 ^b^	1.06 ± 0.01 ^b^	1.08 ± 0.01 ^b^
SR (%)	100 ± 0	100 ± 0	97.5 ± 1.60	100 ± 0	98.34 ± 0.96	99.17 ± 0.83
FCR	1.66 ± 0.06 ^b^	1.42 ± 0.04 ^a^	1.52 ± 0.08 ^ab^	1.40 ± 0.04 ^a^	1.56 ± 0.06 ^ab^	1.52 ± 0.07 ^ab^
CF (g/cm^3^)	1.21 ± 0.02	1.22 ± 0.02	1.16 ± 0.03	1.18 ± 0.04	1.17 ± 0.03	1.17 ± 0.02

Values are presented as mean ± SE, n = 4. Superscript letters in the same row indicate a significant difference at *p* < 0.05.

**Table 7:** The whole-body and muscle composition of *Oncorhynchus mykiss* fed with the experimental diets.

Item	Control	LP	CP	EP	PR	HP
Whole-body, % dry weight						
Moisture	69.24 ± 0.45	68.74 ± 0.71	69.99 ± 1.02	70.64 ± 0.42	69.31 ± 0.34	69.93 ± 0.40
Crude protein	55.17 ± 0.65 ^a^	59.71 ± 0.38 ^b^	59.03 ± 1.03 ^b^	59.07 ± 1.13 ^b^	56.64 ± 0.17 ^ab^	58.71 ± 0.99 ^b^
Crude lipid	33.48 ± 0.48 ^c^	29.39 ± 0.15 ^b^	28.12 ± 0.21 ^a^	28.19 ± 0.20 ^a^	30.41 ± 0.63 ^b^	26.98 ± 0.22 ^a^
Dorsal muscle, % dry weight						
Moisture	78.30 ± 0.36	76.84 ± 0.35	78.62 ± 0.30	76.62 ± 0.77	75.96 ± 0.63	75.96 ± 0.63
Crude protein	75.97 ± 0.68 ^a^	80.44 ± 0.31 ^c^	81.54 ± 0.44 ^c^	79.97 ± 0.68 ^bc^	77.96 ± 0.34 ^b^	77.96 ± 0.34 ^b^
Crude lipid	9.49 ± 0.58	8.74 ± 0.60	8.98 ± 0.63	8.58 ± 0.37	8.61 ± 0.89	8.61 ± 0.89

Values are presented as mean ± SE, n = 12. Superscript letters in the same row indicate a significant difference at *p* < 0.05.

**Table 8:** Amino acid composition in the dorsal muscle of *Oncorhynchus mykiss* fed on the experimental diets (% dry matter).

	Control	LP	CP	EP	PR	HP
Methionine (Met) *	2.32 ± 0.03 ^a^	2.31 ± 0.06 ^a^	2.42 ± 0.04 ^ab^	2.36 ± 0.02 ^ab^	2.48 ± 0.05 ^b^	2.44 ± 0.06 ^ab^
Lysine (Lys) *	6.50 ± 0.07 ^a^	6.52 ± 0.12 ^a^	6.87 ± 0.13 ^ab^	6.57 ± 0.09 ^ab^	6.96 ± 0.12 ^b^	6.83 ± 0.19 ^ab^
Threonine (Thr) *	3.16 ± 0.04 ^a^	3.16 ± 0.06 ^a^	3.31 ± 0.04 ^ab^	3.29 ± 0.05 ^ab^	3.47 ± 0.07 ^b^	3.45 ± 0.07 ^b^
Phenylalanine (Phe) *	3.07 ± 0.03 ^ab^	3.08 ± 0.07 ^ab^	3.32 ± 0.02 ^c^	3.00 ± 0.04 ^a^	3.20 ± 0.06 ^bc^	3.14 ± 0.06 ^ab^
Isoleucine (Ile) *	3.25 ± 0.03 ^a^	3.26 ± 0.06 ^a^	3.40 ± 0.04 ^ab^	3.30 ± 0.03 ^a^	3.48 ± 0.05 ^b^	3.43 ± 0.09 ^ab^
Leucine (Leu) *	5.73 ± 0.05 ^ab^	5.77 ± 0.12 ^ab^	6.05 ± 0.07 ^b^	5.57 ± 0.07 ^a^	5.94±0.12^b^	5.86 ± 0.15 ^ab^
Valine (Val) *	3.89 ± 0.04 ^a^	3.89 ± 0.06 ^a^	4.12 ± 0.06 ^b^	3.83 ± 0.03 ^a^	4.01 ± 0.06 ^ab^	3.99 ± 0.10 ^ab^
Histidine (His) *	2.88 ± 0.05 ^b^	2.92 ± 0.02 ^b^	3.09 ± 0.06 ^c^	1.91 ± 0.05 ^a^	2.01 ± 0.02 ^a^	1.98 ± 0.03 ^a^
Arginine (Arg) *	4.24 ± 0.07 ^ab^	4.14 ± 0.06 ^a^	4.40 ± 0.08 ^ab^	4.14 ± 0.07 ^a^	4.44 ± 0.09 ^b^	4.40 ± 0.12 ^ab^
Glycine (Gly) **	3.69 ± 0.10 ^bc^	3.39 ± 0.06 ^a^	3.78 ± 0.09 ^c^	3.49 ± 0.03 ^ab^	3.63 ± 0.05 ^bc^	3.71 ± 0.09 ^bc^
Serine (Ser) **	2.76 ± 0.04 ^a^	2.74 ± 0.05 ^a^	2.91 ± 0.03 ^ab^	2.82 ± 0.03 ^a^	3.01 ± 0.08 ^b^	3.01 ± 0.06 ^b^
Proline (Pro) **	2.35 ± 0.12 ^ab^	2.23 ± 0.02 ^a^	2.47 ± 0.06 ^b^	2.29 ± 0.02 ^ab^	2.35 ± 0.05 ^ab^	2.36 ± 0.01 ^ab^
Alanine (Ala) **	4.35 ± 0.05 ^ab^	4.32 ± 0.08 ^ab^	4.57 ± 0.07 ^b^	4.27 ± 0.06 ^a^	4.49 ± 0.07 ^ab^	4.42 ± 0.11 ^ab^
Aspartic acid (Asp) **	7.48 ± 0.09 ^ab^	7.47±0.14 ^ab^	7.85 ± 0.06 ^b^	7.28 ± 0.08 ^a^	7.71 ± 0.13 ^ab^	7.59 ± 0.06 ^ab^
Glutamic acid (Glu) **	10.00 ± 0.14 ^a^	10.02 ± 0.19 ^ab^	10.66 ± 0.18 ^b^	10.12 ± 0.14 ^a^	10.62 ± 0.20 ^b^	10.54 ± 0.29 ^ab^
Essential amino acids	35.04 ± 0.40 ^ab^	35.04 ± 0.62 ^ab^	36.99 ± 0.45 ^b^	33.96 ± 0.45 ^a^	36.01 ± 0.63 ^ab^	35.52 ± 0.87 ^ab^
Non-essential amino acids	30.77 ± 0.47	30.17 ± 0.52	32.23 ± 0.47	30.28 ± 0.30	31.81 ± 0.58	31.63 ± 0.75
Total amino acids	65.81 ± 0.85	65.21 ± 1.14	69.22 ± 0.92	64.24 ± 0.74	67.81 ± 1.21	67.15 ± 1.62

* essential amino acid; ** non-essential amino acids. Values are presented as mean ± SE, n = 4. Superscript letters in the same row indicate a significant difference at *p* < 0.05.

## Data Availability

The data that support the findings of this study are available from the corresponding author upon reasonable request.
